# Supplementary material for: An engineered ligand trap inhibits leukemia inhibitory factor as pancreatic cancer treatment strategy
Source: Commun Biol. 2021 Apr 12;4:452. doi: 10.1038/s42003-021-01928-2 (PMC8041770; doi:10.1038/s42003-021-01928-2)
Supplement: Supplementary file 2 — Supplementary Information [file 42003_2021_1928_MOESM2_ESM.pdf]

Supplementary Material

An engineered ligand trap inhibits leukemia inhibitory factor as pancreatic cancer treatment strategy

Sean A. Hunter[1], Brianna J. McIntosh[1], Yu Shi[2], R. Andres Parra Sperberg[3], Chie Funatogawa[4], Louai Labanieh[3], Erin Soon[5], Hannah C. Wastyk[3], Nishant Mehta[3], Catherine Carter[3], Tony Hunter[2], & Jennifer R. Cochran[1,3,5,6]*

[1]Cancer Biology Program, Stanford University School of Medicine, Stanford, California 94305, USA

[2]Molecular and Cell Biology Laboratory, Salk Institute for Biological Studies, La Jolla, California 92037, USA

[3]Department of Bioengineering, Stanford University, Stanford, California 94305, USA

[4]Unchained Labs, Pleasanton, California 94566, USA

[5]Immunology Program, Stanford University School of Medicine, Stanford, California 94305, USA

[6]Department of Chemical Engineering, Stanford University, Stanford, California 94305, USA

*To whom correspondence should be addressed: jennifer.cochran@stanford.edu

Supplementary Fig. 1: mLIFR-Fc inhibits LIF signaling.

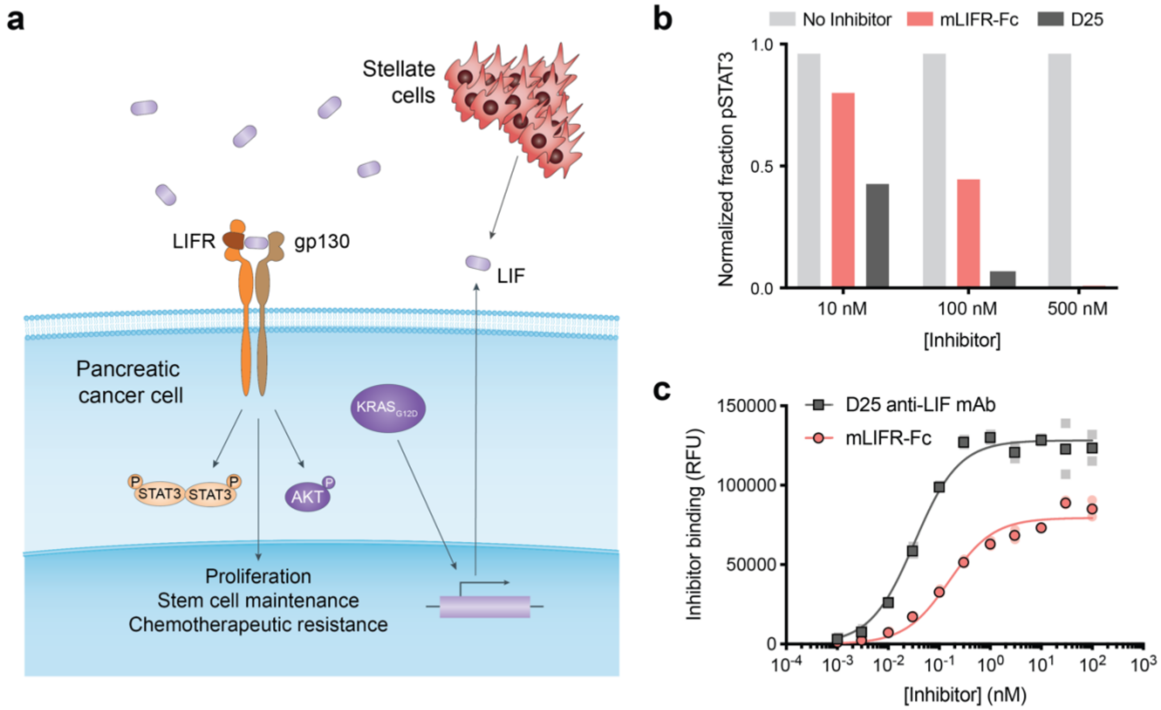

a LIF is produced both from activated myofibroblastic stellate cells and from PDAC cells in the tumor microenvironment. LIF signals through the heterodimeric receptors LIFR and gp130. **b** Quantification of the pSTAT3 signal in the immunoblot shown in Fig. 1b reveals that mLIFR-Fc inhibits pSTAT3. Data are the mean relative intensity of the pSTAT3 signal normalized to the relative intensity from tubulin staining. **c** Binding curve of mLIFR-Fc (apparent K_d = 160 ± 20 pM; pink circles) and the anti-LIF D25 mAb (apparent K_d = 33 ± 8 pM; gray squares) to yeast surface-displayed mLIF. Curves were fit and K_d values calculated using non-linear regression. Data are the mean of fluorescence of the expressing population (n = 2).

Supplementary Table 1: Blood analysis reveals no toxicity from mLIFR-Fc treatment.

	FVB		Black/6		
**Analyte**	**Control**	**Treated**	**Control**	**Treated**	**Units**
WBC	6.69 ± 0.892	8.544 ± 2.418	4.98 ± 1.123	5.958 ± 1.558	K/uL
RBC	10.33 ± 0.605	10.808 ± 0.343	11.08 ± 0.587	10.952 ± 0.605	M/uL
HGB	15.17 ± 0.907	15.9 ± 0.731	16.1 ± 0.854	16.04 ± 0.716	gm/dL
HCT	50.47 ± 2.23	53.28 ± 1.445	55.17 ± 2.71	55.7 ± 2.519	%
MCV	48.9 ± 0.7	49.32 ± 0.356	49.8 ± 0.265	50.88 ± 0.963	fL
MCH	14.67 ± 0.058	14.72 ± 0.192	14.53 ± 0.058	14.66 ± 0.336	pg
MCHC	30.03 ± 0.493	29.82 ± 0.61	29.17 ± 0.115	28.8 ± 0.332	g/dL
Platelet Count	1480.7 ± 203.8	1563.6 ± 216	1459.3 ± 117.9	1489.2 ± 200.4	K/uL
RDW	20.8 ± 0.866	21.28 ± 0.449	20.13 ± 0.586	20.08 ± 0.698	%
PDW	11.37 ± 0.902	10.44 ± 0.74	7.57 ± 0.153	7.86 ± 0.288	fL
MPV	8.03 ± 0.404	7.7 ± 0.292	6.33 ± 0.058	6.58 ± 0.239	fL
P-LCR	15.67 ± 2.701	13.14 ± 2.04	3.6 ± 0.2	4.66 ± 1.085	%
PCT	1.19 ± 0.227	1.206 ± 0.195	0.93 ± 0.074	0.984 ± 0.146	%
Reticulocyte Count	4.8 ± 0.201	4.92 ± 0.126	4.69 ± 0.609	4.9 ± 0.547	%
IRF	47.8 ± 1.277	50.26 ± 1.297	51.73 ± 0.862	52.46 ± 2.439	%
IFR	52.2 ± 1.277	49.76 ± 1.299	48.27 ± 0.862	47.54 ± 2.439	%
MFR	15.9 ± 1.058	17.94 ± 0.451	25.33 ± 1.305	24.24 ± 1.212	%
Reticulocyte Abs.	496466 ± 50097	532061 ± 30023	517547 ± 51272	537311 ± 73936	/uL
Platelet Estimate	Adequate	Adequate	Adequate	Adequate	K/uL
Neutrophils	3 ± 1.732	7.6 ± 2.074	4.33 ± 2.082	5.6 ± 1.673	%
Lymphocytes	94 ± 1	80.6 ± 5.225	90.67 ± 4.041	85.8 ± 7.53	%
Monocytes	2.67 ± 1.155	8 ± 3.536	4.33 ± 1.528	7.8 ± 5.263	%
Eosinophils	0.33 ± 0.577	3.8 ± 2.588	0.67 ± 0.577	0.8 ± 1.304	%
Basophils	0 ± 0	0 ± 0	0 ± 0	0 ± 0	%
RBC Morphology	Normal	Normal	Normal	Normal	
Glucose	246.67 ± 63.12	244.8 ± 36.68	261.67 ± 19.09	253.75 ± 42.24	mg/dL
AST	80.33 ± 10.12	145.4 ± 73.57	148.33 ± 35.25	91.8 ± 23.75	U/L
ALT	57 ± 14	71.4 ± 24.72	41.33 ± 9.292	28.4 ± 2.702	U/L
Alkaline Phosphatase	153.33 ± 12.06	168.2 ± 31.35	133.67 ± 104.5	202.4 ± 15.96	IU/L
GGT	0 ± 0	0 ± 0	0 ± 0	0 ± 0	U/L
Total Bilirubin	0.37 ± 0.058	0.3 ± 0.071	0.43 ± 0.153	0.325 ± 0.096	mg/dL
Cholesterol	179 ± 12.29	186.8 ± 25.12	83.5 ± 0.707	81 ± 15.47	mg/dL
BUN	18.33 ± 1.528	21.8 ± 3.633	18.67 ± 2.517	20.4 ± 2.881	mg/dL
Creatinine	0.17 ± 0.012	0.194 ± 0.07	0.09 ± 0.101	0.2 ± 0.086	mg/dL
Calcium	10.7 ± 0.436	10.86 ± 0.477	9.87 ± 0.451	10.97 ± 0.306	mg/dL
Phosphorus	11.7 ± 0.173	11.5 ± 0.557	11.07 ± 0.862	12.25 ± 0.58	mg/dL
T.Protein	6.1 ± 0.2	6.2 ± 0.2	5.9 ± 0.1	5.8 ± 0.245	g/dL
Albumin	3.57 ± 0.153	3.58 ± 0.148	3.77 ± 0.115	3.7 ± 0.141	g/dL
Globulin	2.53 ± 0.058	2.62 ± 0.13	2.13 ± 0.153	2.1 ± 0.115	
Triglycerides	567 ± 14.14	463.2 ± 89.74	91.67 ± 9.074	76 ± 0	mg/dL
CK	160.5 ± 55.86	317.25 ± 206.8	273 ± 96.17	178 ± 0	U/L

Summary of analytes identified from the serum of FVB and Black/6 mice after one month of mLIFR-Fc injection (IP; 3x/week; 20 mg/kg). n=3 for the “Control” PBS injected group and n=5 for the mLIFR-Fc “Treated” group for each species.

Supplementary Fig. 2: LIFR Ig-like domain characterization, library generation, and screening.

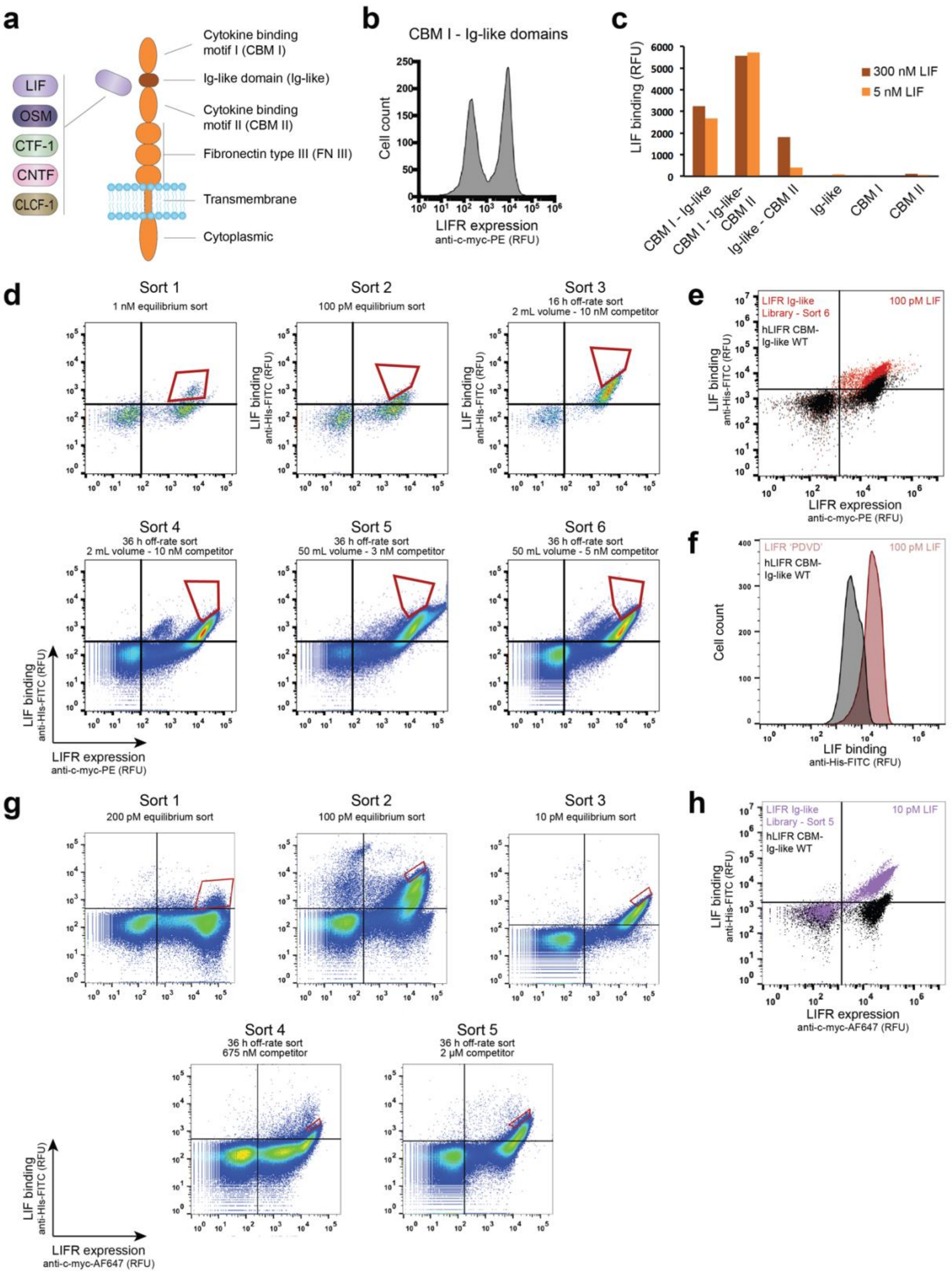

a Schematic of LIFR domains and binding partners. LIF interacts with LIFR mainly through the extracellular Ig-like domain. **b** LIFR domain combination of CBM I – Ig-like expresses well when displayed on the surface of yeast as analyzed by flow cytometry. **c** Binding of various yeast-displayed LIFR domain combinations to 300 nM and 5 nM LIF. Data are the geometric mean of fluorescence minus baseline where no LIF was added. **d** Library 1: Fluorescence-activated cell sorting (FACS) dot plots and screening gates (red polygons). Each collected yeast population was used for the next sort. **e** Flow cytometry dot plot of the yeast pool remaining after six rounds of sorting (red) shows increased LIFR binding to 100 pM LIF as compared to WT LIFR (black). **f** Flow cytometry histogram showing that LIFR L218P–N242D–I257V–N277D (‘PDVD’), the most frequent variant identified from Library 1 (red) has improved binding to 100 pM LIF over WT hLIFR (black). **g** Library 2: FACS dot plots and screening gates (red polygons). **h** Flow cytometry dot plot of the yeast pool remaining after 5 rounds of sorting (purple) shows increased LIFR binding to 10 pM LIF as compared to WT LIFR (black).

Supplementary Fig. 3: Characterization of soluble, recombinant LIFR-Fc fusion proteins.

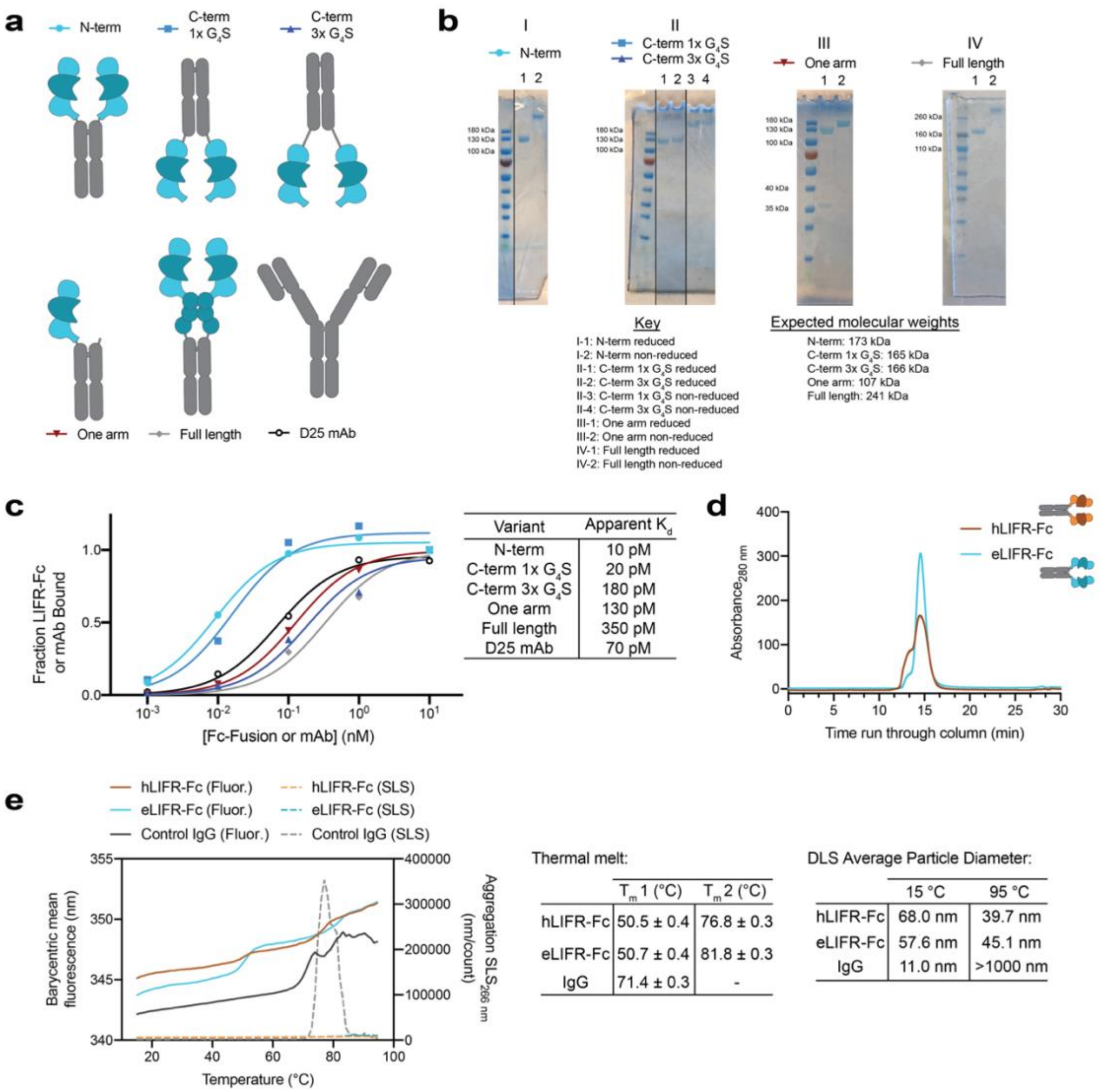

a Soluble versions of eLIFR constructed and tested for LIF binding affinity. Gray, Fc-domain; blue, LIFR domains. Schematic of the anti-LIF mAb, D25 is also shown. 1x G_4S = $(Gly)_4Ser$ linker; 3x G_4S = $[(Gly)_4Ser]_3$ linker. **b** Representative gel images of purified Fc-fusions analyzed as reduced and non-reduced samples on SDS-PAGE. Lanes were taken from the same gel and have now been placed side-by-side for clarity with lines indicating lanes that were not adjacent on the original gel. Gels of purified proteins were run at least three times with similar results. **c** N-terminal and C-terminal 1x G_4S eLIFR-Fc fusions have the strongest binding affinity to yeast-displayed LIF. Key: N-terminal (light blue circles), C-terminal 1x G_4S linker (blue squares), C-terminal 3x G_4S linker (purple triangles), one-arm heterodimeric (red inverted triangles), full-length LIFR (gray diamonds), and anti-LIF mAb D25 (black open circles). Binding curves were fit using non-linear regression to generate apparent K_d values. **d** Representative size exclusion chromatography plots of eLIFR-Fc and hLIFR-Fc run on FPLC. Proteins were run three times with similar results. **e** Melting and aggregation temperature analysis hLIFR-Fc, eLIFR-Fc, and a control IgG (dinutuximab) quantified by barycentric mean of fluorescence, SLS, and DLS. Data are the mean (n=3).

Supplementary Fig. 4: eLIFR-Fc blocks LIF signaling in HeLa STAT3 luciferase reporter cells.

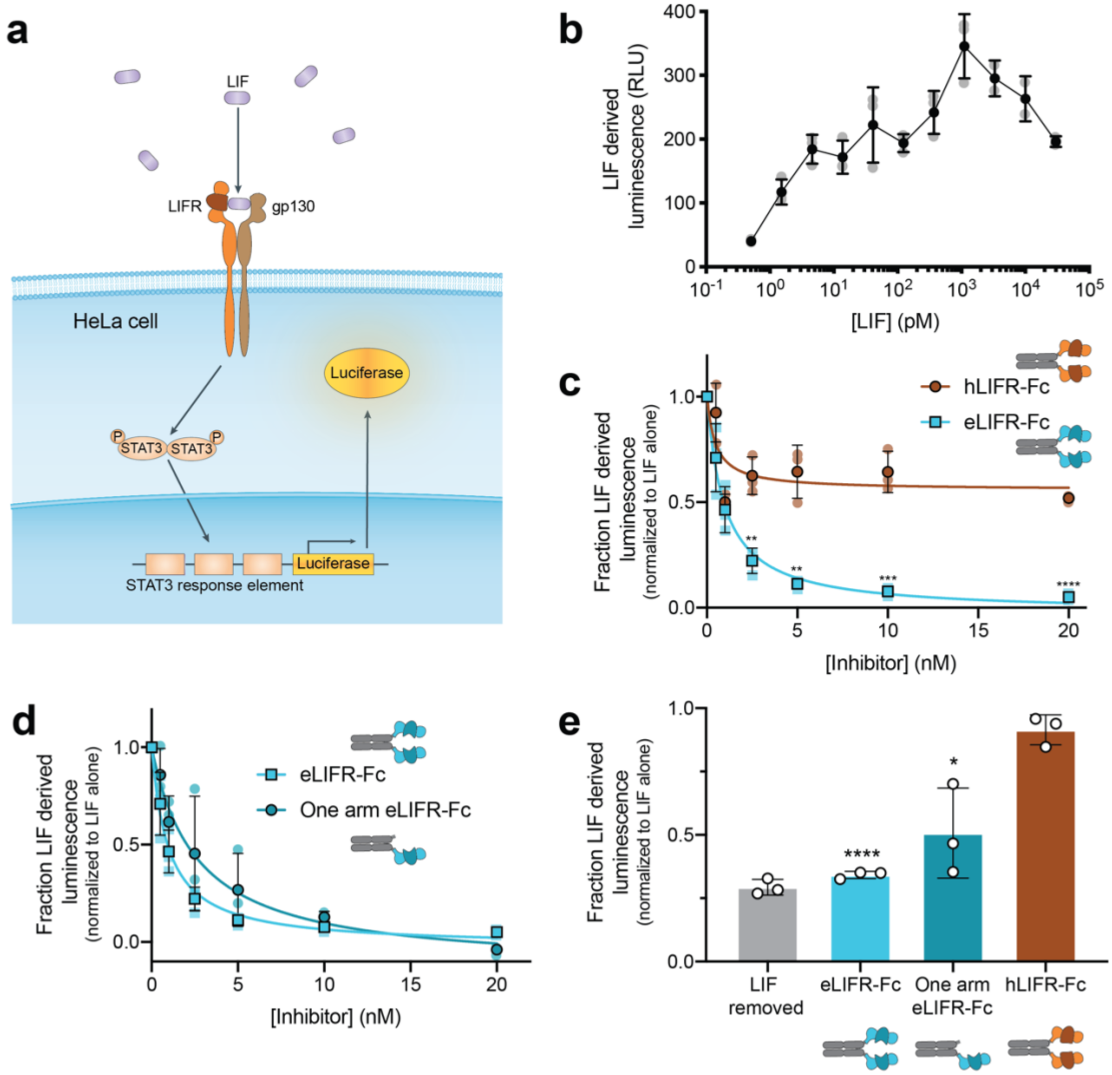

a LIF binding to LIFR and gp130 drives phosphorylation and subsequent dimerization of STAT3, which enters the nucleus and transcriptionally activates a triple STAT3 response element. This element precedes the firefly luciferase gene, thus linking LIF signaling with luciferase expression. **b** Luciferase signal peaks at 1 nM LIF in a dose response of HeLa reporter cells from 0.5 pM to 30 nM LIF. Data are the mean ± SD (n = 3). **c** eLIFR-Fc (blue squares) nearly completely silences LIF signaling, while hLIFR-Fc (brown circles), with no competitive LIF binding advantage, is ineffective at silencing LIF signaling, even at concentrations 40-fold in excess. Normalized luminescence from HeLa reporter cells in response to 0.5 nM LIF, incubated overnight with the indicated concentrations of eLIFR-Fc or hLIFR-Fc. ***P* = 0.002, ****P* = 0.0006, *****P* < 0.0001 versus the corresponding hLIFR-Fc-treated condition, two-tailed unpaired Student's *t*-test. Data are the mean luminescence minus baseline (no LIF added), normalized to signal without inhibitor, ± SD (n = 3). **d** Both eLIFR-Fc (light blue squares) and one-arm eLIFR-Fc (teal circles) potently inhibit LIF signaling. Normalized luminescence from HeLa reporter cells in response to 0.5 nM LIF, incubated with the indicated concentrations of constructs for 5 h. Data are the mean luminescence minus baseline, normalized to signal without added inhibitor, ± SD (n = 3). **e** LIFR constructs differentially block signaling when added in a delayed fashion. Normalized luminescence from HeLa reporter cells incubated with 0.5 nM LIF for 1 h, followed by addition of 5 nM of the indicated construct or fresh medium ("LIF removed"). Cells were left for 20 h before luminescence was quantified. Raw luminescence, minus baseline luminescence, was normalized to the luminescence from the condition without inhibitor. **P* = 0.02, *****P* < 0.0001 compared to corresponding hLIFR-Fc condition by two-tailed unpaired Student's *t*-test. Data are the mean ± SD (n = 3).

Supplementary Fig. 5: eLIFR-Fc blocks LIF activity in PDAC cells.

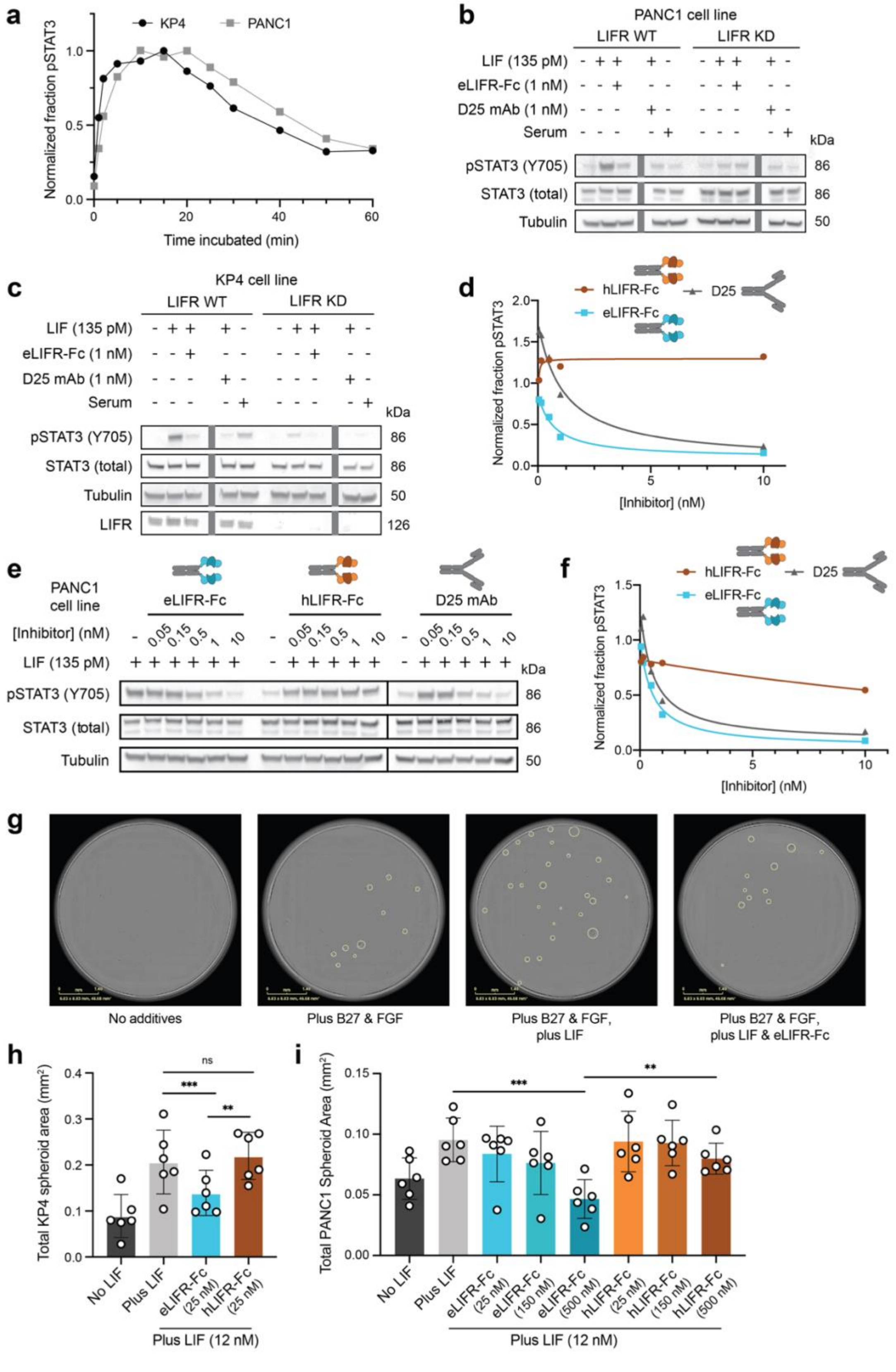

a Time course of pSTAT3 signal, derived from LIF incubation with KP4 (black circles) or PANC1 (grey squares) cells over 60 min. Data are the mean intensity of an immunoblot against pSTAT3, normalized to the highest pSTAT3 signal observed, quantified using ImageJ. **b,c** Immunoblotting of lysates from **b** PANC1(LIFR WT and knockdown (KD)) and **c** KP4 (LIFR WT and KD) cells treated with LIF and/or eLIFR-Fc or D25. Tubulin served as the loading control. Serum, 10% fetal bovine serum. Grey lines indicate removed lanes. **d** Quantification of pSTAT3 signal in KP4 cells (**Fig. 4c**). Curves fit with non-linear regression. Data are the mean relative intensity of the pSTAT3 signal normalized to the relative intensity from tubulin and total STAT3 staining. **e** Immunoblots of cell lysates from PANC1 cells incubated with LIF (0.135 nM) and/or eLIFR-Fc, hLIFR-Fc or the D25 mAb. Immunoblotting is as described in panel **b**. Lines indicate lanes that were not run adjacently. **f** Quantification of pSTAT3 signal from panel **e**, performed as described in panel **d**. **g** Representative Incucyte whole 96-well images of 2 weeks of spheroid growth (yellow circles) of KP4 cells in conditions with DMEM/F12 + methylcellulose alone, or with additives ([B27] = 50 ng/mL; [FGF] = 20 ng/mL; [LIF] = 12 nM; [eLIFR-Fc] = 500 nM). **h** Quantification of total sphere area of KP4 cells after 1 week of growth and treatment with 25 nM eLIFR-Fc or hLIFR-Fc. [LIF] = 12 nM. $^{**}P = 0.0034$, $^{***}P = 0.0006$ versus “plus LIF” or between LIFR-Fc treatments, as indicated, by two-tailed unpaired Student’s *t*-test. Data are the mean total sphere area ± SD (n = 6). **i** Quantification of total sphere area in PANC1 cells after 2 weeks of growth, treated with 25 nM, 150 nM, or 500 nM eLIFR-Fc or hLIFR-Fc. [LIF] = 12 nM. $^{**}P = 0.0025$, $^{***}P = 0.0006$ compared to “plus LIF” or between LIFR-Fc treatments, as indicated, by two-tailed unpaired Student’s *t*-test Data are the mean total sphere area ± SD (n = 6).

Supplementary Fig. 6: eLIFR-Fc binds to and inhibits LIF more strongly compared to other IL-6 family cytokines.

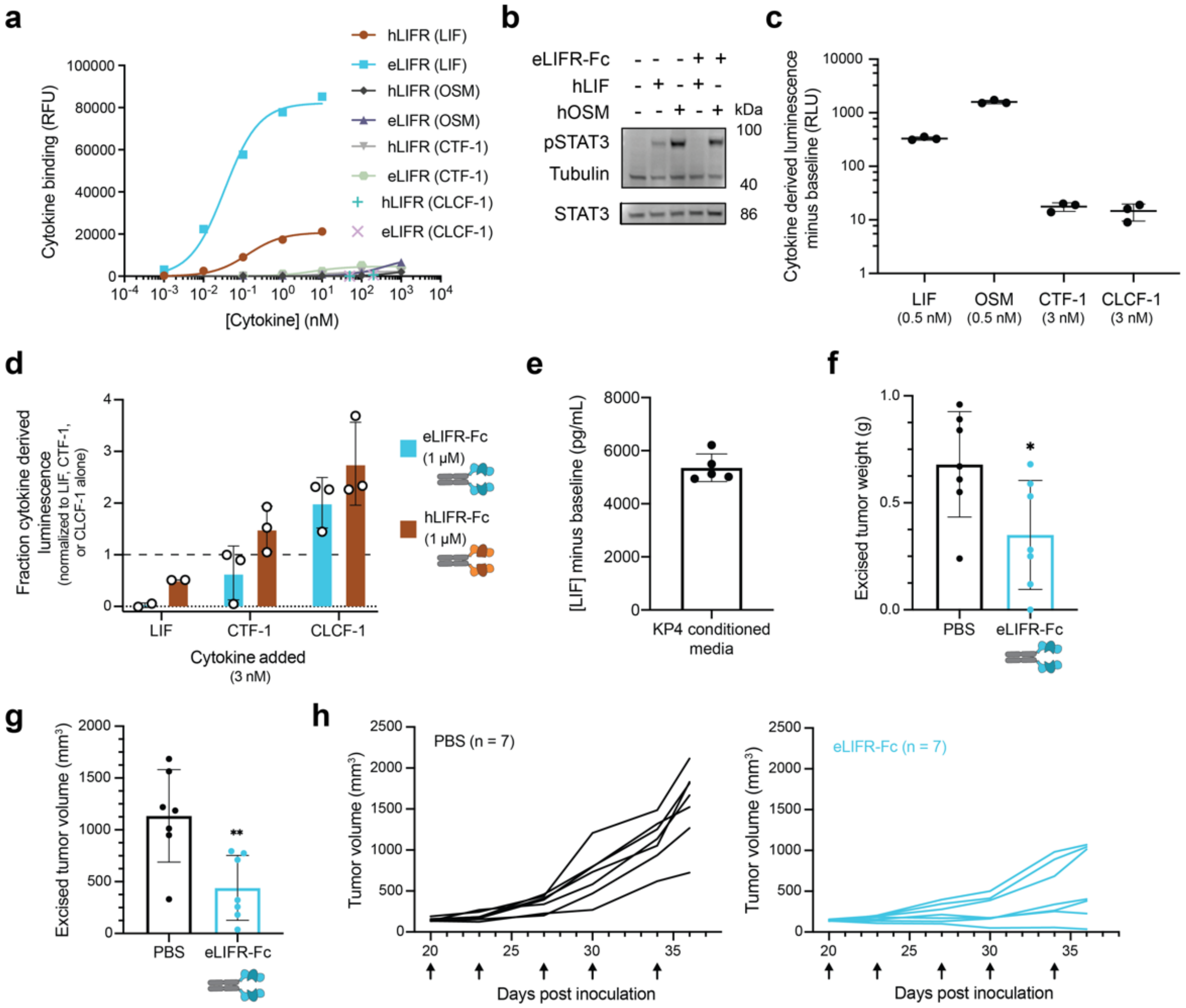

a Binding of yeast-displayed eLIFR (light blue squares) and hLIFR (brown circles) to LIF. In comparison, negligible binding was observed to related cytokines OSM (purple triangles, dark gray diamonds), CTF-1 (green hexagons, light gray inverted triangles), or CLCF-1 (pink X, teal +). Data are the mean of fluorescence of the expressing population minus the fluorescence of the baseline condition where no cytokine was added. **b** In KP4 PDAC cells, coincubation of 20 nM eLIFR-Fc with 0.5 nM OSM yields minimal inhibition, in contrast to full inhibition with 0.5 nM LIF. Immunoblot of lysates from cells treated as indicated. Tubulin serves as the loading control. **c** Magnitude of luminescence from HeLa STAT3 reporter cells differs in response to treatment with IL-6 family cytokines, indicating a more robust signal from LIF and OSM as compared to CTF-1 or CLCF-1. [LIF] = 0.5 nM; [OSM] = 0.5 nM; [CTF-1-Fc] = 3 nM; [CLCF-1] = 3 nM. Data are the mean of the raw luminescence minus baseline ± standard deviation (n = 3). **d** High concentrations of eLIFR-Fc or hLIFR-Fc minimally inhibit or possibly promote mild agonism of CTF-1 or CLCF-1 signaling in HeLa STAT3 cells as compared to LIF. Raw luminescence, minus baseline luminescence, was normalized to the luminescence from the respective condition without inhibitor (dashed line). Data are the mean ± SD (n=3 for all except 3 nM LIF, where n=2). **e** LIF concentration in conditioned media collected from KP4 cells, quantified by ultra-sensitive ELISA, minus the baseline [LIF] in media alone. Data are the mean ± SD (n = 5). **f** Weights of excised KP4 treated tumors. *P=0.03 versus PBS treated tumors by two-tailed unpaired Student's t-test. Data are the mean ± SD (n = 7). **g** Volumes of excised KP4 treated tumors. **P=0.006 versus PBS treated tumors by two-tailed unpaired Student's t-test. Data are the mean ± SD (n = 7). **h** Tumor measurements from individual mice in KP4 study (PBS treated, left; eLIFR-Fc treated, right). Dosing schedule indicated by arrows.

Supplementary Fig. 7: Modeling eLIFR binding to hLIF.

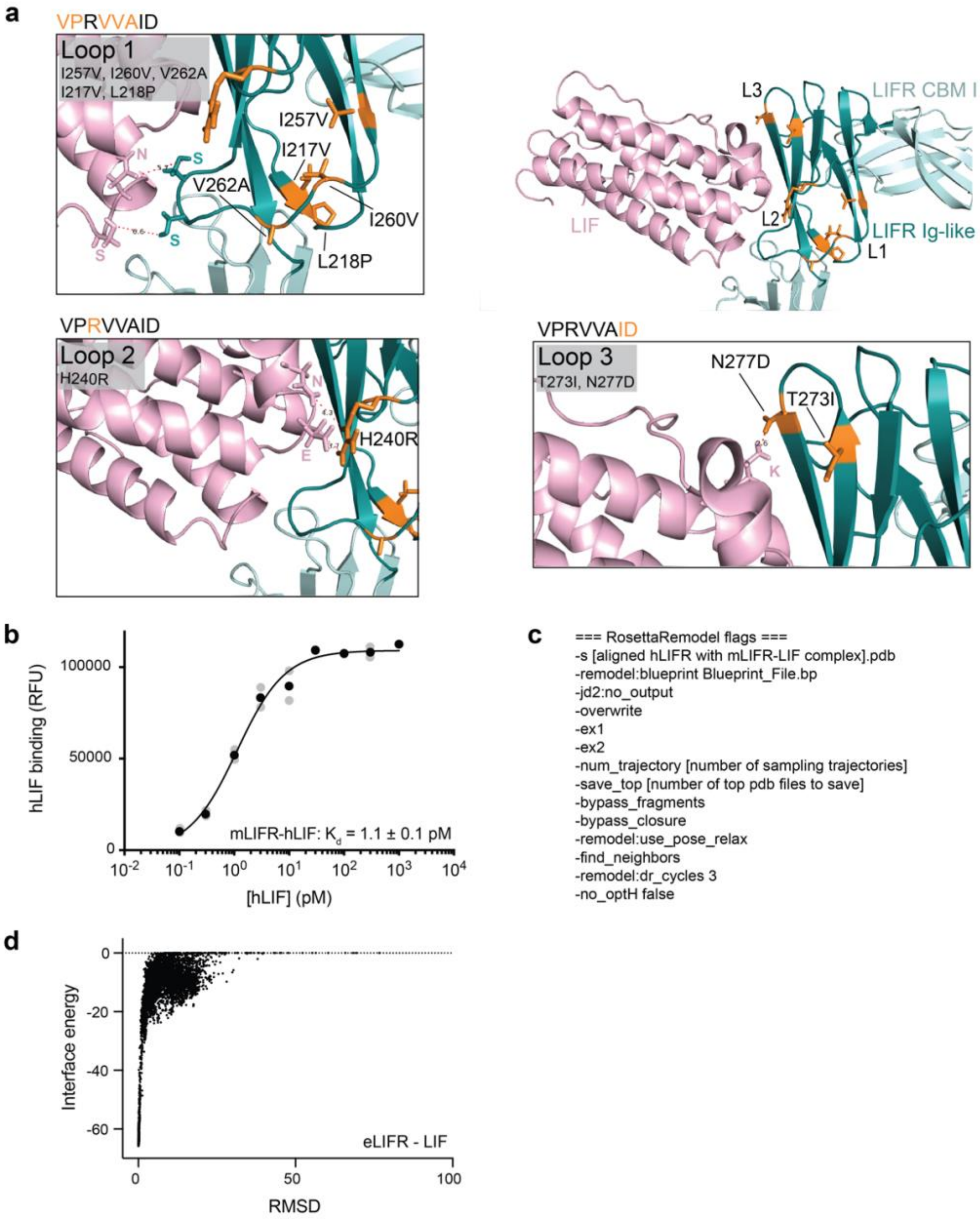

a Proposed structure of eLIFR in complex with LIF, modeled by inserting mutations into hLIFR WT PDB (3E0G) using Rosetta Remodel (Materials and Methods). hLIF was aligned locally to the eLIFR structure using Rosetta Dock with excellent convergence. "VPRVVAID" mutations are shown in orange. Three loop regions of interest are labeled L1, L2, and L3, with mutations in orange. Likely interacting residues in LIF are pink, with distances measured in PyMOL in red (dashed lines). **b** Yeast-displayed mLIFR binds hLIF with an apparent affinity of 1.1 ± 0.1 pM. Curves were fit and K_d calculated using non-linear regression. Data are the mean of fluorescence of the expressing population (n = 2). **c** Flags used during execution of the Rosetta Remodel program in order to model the eLIFR-hLIF interaction. **d** Funnel plot of the Rosetta local docking results for the eLIFR – LIF interaction. All 10,000 simulated docking interactions are shown. RMSD, root mean square deviation.

Supplementary Fig. 8: Uncropped blots and gels.

Fig. 1b

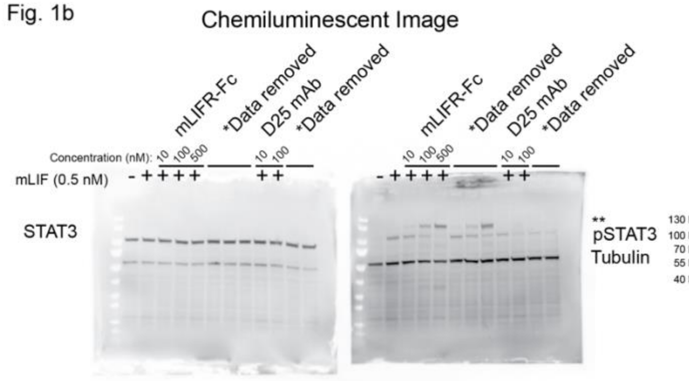

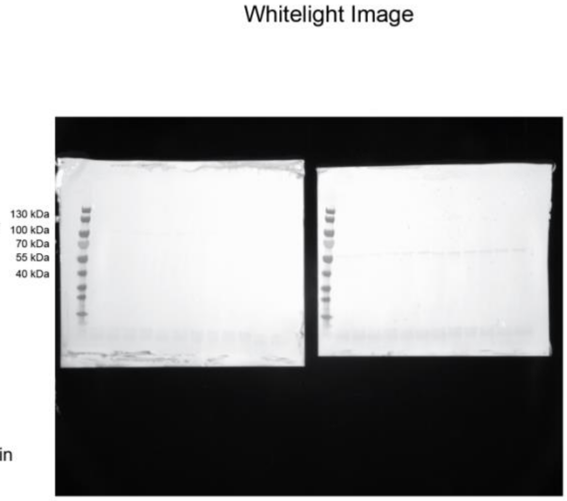

*Data removed from figure as they describe non-relevant protein

** Bands above pSTAT3 are residual mLIFR-Fc in the lysate prep; detected as a by-product of the anti-mouse antibody used to detect Tubulin

Fig. 4c

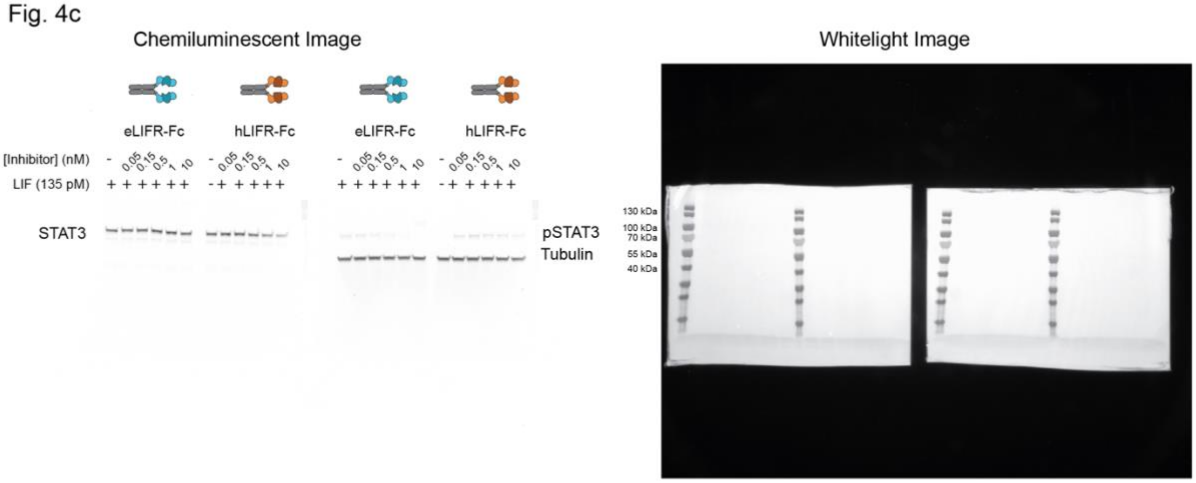

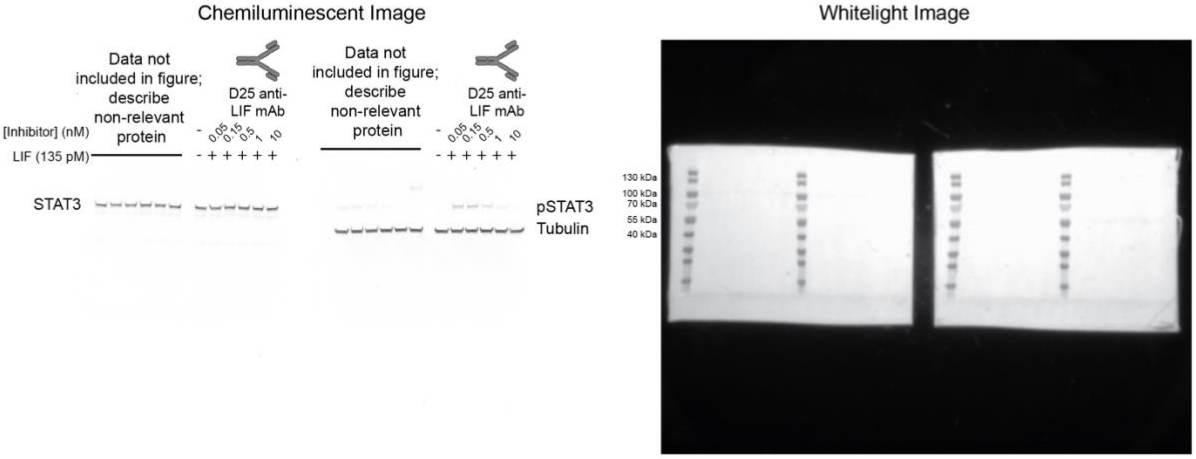

Gels from Supplementary Figure 4b

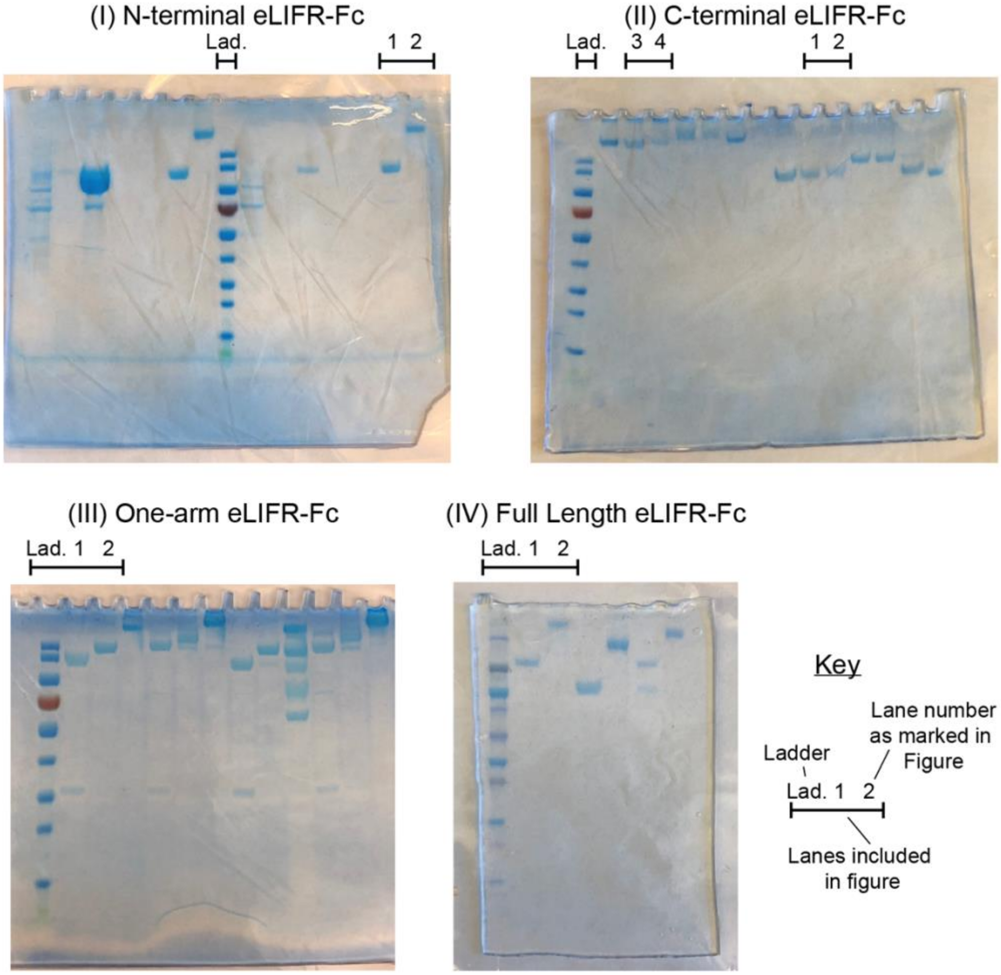

Supplementary Fig. 5b

* : Lanes removed from blots in figure; describe non-relevant protein

Chemiluminescent Image

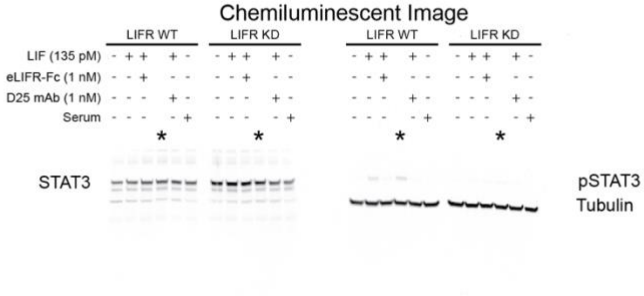

Whitelight Image

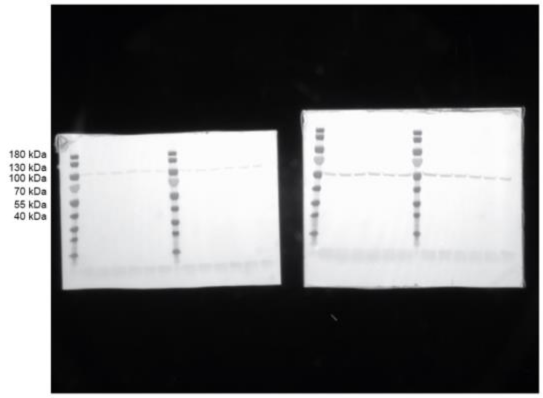

Supplementary Fig. 5c

Chemiluminescent Image

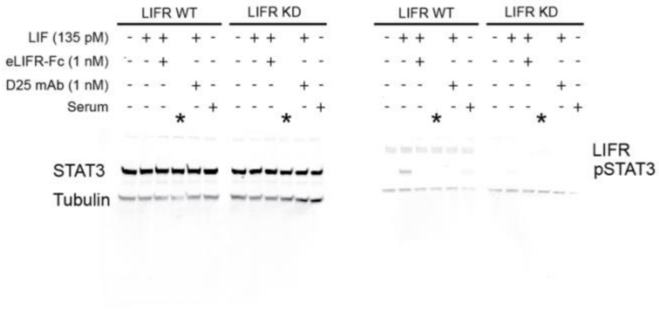

Whitelight Image

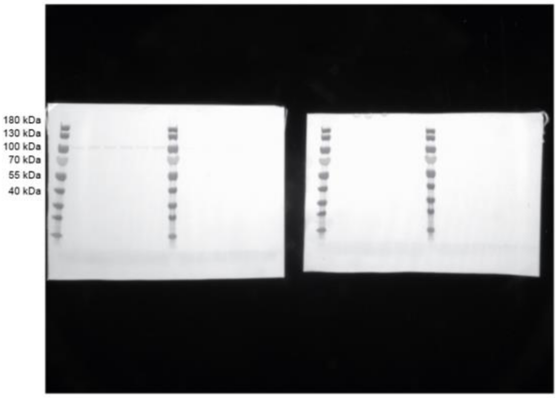

Supplementary Fig. 5e

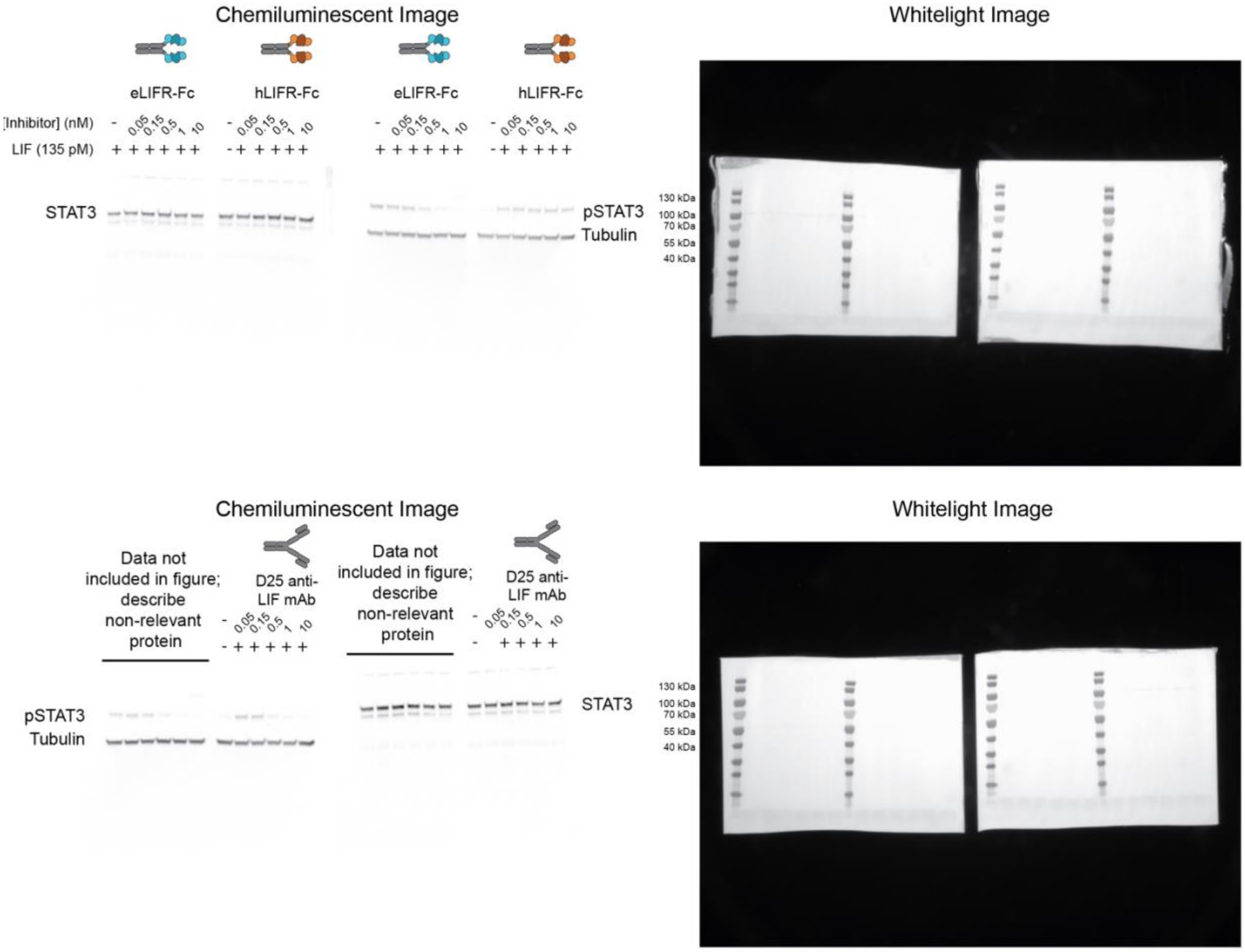

Supplementary Fig. 6b

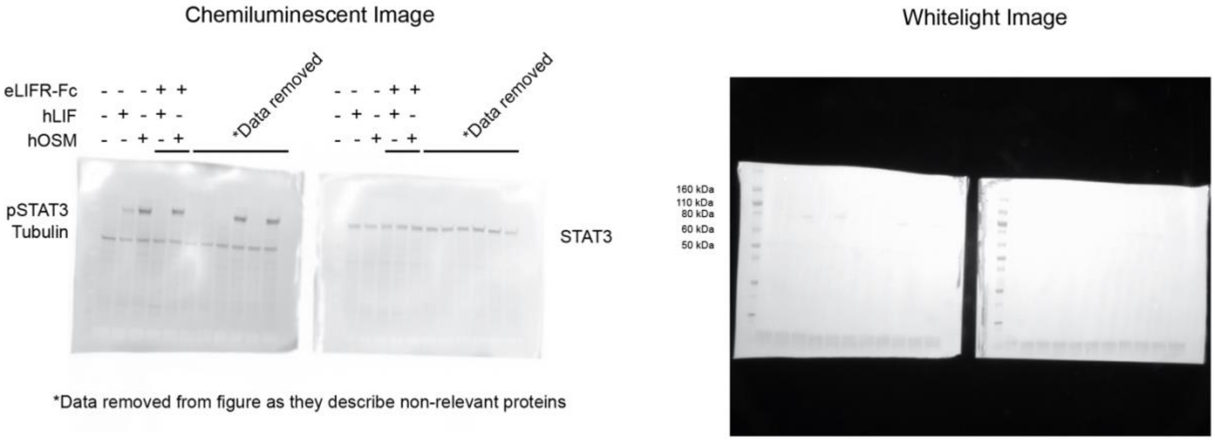

*Data removed from figure as they describe non-relevant proteins
