# Supplementary material for: An engineered ligand trap inhibits leukemia inhibitory factor as pancreatic cancer treatment strategy
Source: Commun Biol. 2021 Apr 12;4:452. doi: 10.1038/s42003-021-01928-2 (PMC8041770; doi:10.1038/s42003-021-01928-2)
Supplement: Supplementary file 3 — Description of Additional Supplementary Files [file 42003_2021_1928_MOESM3_ESM.pdf]

Description of Additional Supplementary Files

File name: Supplementary Data 1

Description: Source data for graphical representations in Figures 1 – 4.
