# Supplementary material for: An engineered ligand trap inhibits leukemia inhibitory factor as pancreatic cancer treatment strategy
Source: Commun Biol. 2021 Apr 12;4:452. doi: 10.1038/s42003-021-01928-2 (PMC8041770; doi:10.1038/s42003-021-01928-2)
Supplement: Supplementary file 5 — Reporting Summary [file 42003_2021_1928_MOESM5_ESM.pdf]

nature research

Corresponding author(s): Jennifer R. Cochran

Last updated by author(s): Dec 23, 2020

Reporting Summary

Nature Research wishes to improve the reproducibility of the work that we publish. This form provides structure for consistency and transparency in reporting. For further information on Nature Research policies, see Authors & Referees and the Editorial Policy Checklist .

Statistics

For all statistical analyses, confirm that the following items are present in the figure legend, table legend, main text, or Methods section.

n/a	Confirmed	
☐	☒	The exact sample size (*n*) for each experimental group/condition, given as a discrete number and unit of measurement
☐	☒	A statement on whether measurements were taken from distinct samples or whether the same sample was measured repeatedly
☐	☒	The statistical test(s) used AND whether they are one- or two-sided<br>*Only common tests should be described solely by name; describe more complex techniques in the Methods section.*
☒	☐	A description of all covariates tested
☐	☒	A description of any assumptions or corrections, such as tests of normality and adjustment for multiple comparisons
☐	☒	A full description of the statistical parameters including central tendency (e.g. means) or other basic estimates (e.g. regression coefficient) AND variation (e.g. standard deviation) or associated estimates of uncertainty (e.g. confidence intervals)
☐	☒	For null hypothesis testing, the test statistic (e.g. *F*, *t*, *r*) with confidence intervals, effect sizes, degrees of freedom and *P* value noted<br>*Give P values as exact values whenever suitable.*
☒	☐	For Bayesian analysis, information on the choice of priors and Markov chain Monte Carlo settings
☒	☐	For hierarchical and complex designs, identification of the appropriate level for tests and full reporting of outcomes
☒	☐	Estimates of effect sizes (e.g. Cohen's *d*, Pearson's *r*), indicating how they were calculated

Our web collection on statistics for biologists contains articles on many of the points above.

Software and code

Policy information about availability of computer code

Data collection	Cells were sorted using BD FACSDiva version 8.0.1 software (Becton, Dickinson and Company). Flow cytometry data was collected using BD Accuri software (Becton, Dickinson and Company). Size exclusion chromatography data was collected on an Agilent 1260 Infinity II Analytical-Scale LC Purification System using a Superdex 200 3.2/300 column. Melting temperature analysis was collected on an Uncle Instrument (Unchained Labs) and analyzed with Uncle Analysis software (Unchained Labs). Kinetic Exclusion Assay data was collected using KinExA Pro version 3.6.2 software (Sapidyne Instruments). Luminescence from HeLa STAT3 luciferase reporter cell assays was collected using Gen2 software (BioTek). Western blot images were collected using Quantity One software (BioRad). Cancer cell spheroid images were collected using Incucyte standard software (Sartorius). For ELISA, signal acquisition and subsequent analysis was carried out by Quanterix SX-R.
Data analysis	Flow cytometry data was analyzed using FlowJo version 10.6.1 (Treestar). Prism version 8.0.2(GraphPad) was used to plot data and used for statistical analysis. Some data was analyzed and plotted in Excel version 16.34 (Microsoft). ImageJ version 1.49 was used for image quantification. PyMOL version 1.7.4.4 (Schrödinger, LLC) was used to analyze and view protein structures. Rosetta was used to model mutations in LIFR and the LIF-LIFR interaction. Kinetic Exclusion Assay binding data was analyzed using KinExA Pro version 3.6.2 software (Sapidyne Instruments). Size exclusion chromatography data was analyzed using Agilent software. Melting temperature analysis was analyzed with Uncle Analysis software (Unchained Labs).

For manuscripts utilizing custom algorithms or software that are central to the research but not yet described in published literature, software must be made available to editors/reviewers. We strongly encourage code deposition in a community repository (e.g. GitHub). See the Nature Research guidelines for submitting code & software for further information.

Data

Policy information about availability of data

All manuscripts must include a data availability statement. This statement should provide the following information, where applicable:

- Accession codes, unique identifiers, or web links for publicly available datasets
- A list of figures that have associated raw data
- A description of any restrictions on data availability

The data that support the findings of this study are available from the corresponding author upon reasonable request.

Field-specific reporting

Please select the one below that is the best fit for your research. If you are not sure, read the appropriate sections before making your selection.

[x] Life sciences   [ ] Behavioural & social sciences   [ ] Ecological, evolutionary & environmental sciences

For a reference copy of the document with all sections, see nature.com/documents/nr-reporting-summary-flat.pdf

Life sciences study design

All studies must disclose on these points even when the disclosure is negative.

Sample size	Cell numbers for in vitro experiments were determined based on the minimum number of cells required for logarithmic growth and were based on previous literature. For western protein analysis 75% confluency was chosen to maximize the extracted protein concentration while avoiding cell contact inhibition. For in vivo studies, as many biologically independent samples as possible were included.
Data exclusions	No data were excluded from analysis
Replication	All attempts at replication were successful. For a majority of the experiments, we performed three independent experiments and assessed for similar results.
Randomization	For the in vivo xenograft study, mice were randomly assigned to treatment groups, after measuring initial tumor size and allocating mice such that mean tumor size was even between treatment groups. For in vivo toxicity studies mice were randomly assigned to treatment groups.
Blinding	During in vivo studies (both xenograft and toxicity), investigators were blinded to conditions. Samples were blinded during identification of spheroids from Incucyte images. All other experiments were not blinded, but samples were treated and analyzed identically, as appropriate.

Reporting for specific materials, systems and methods

We require information from authors about some types of materials, experimental systems and methods used in many studies. Here, indicate whether each material, system or method listed is relevant to your study. If you are not sure if a list item applies to your research, read the appropriate section before selecting a response.

Materials & experimental systems

n/a	Involved in the study	
[ ]	[x]	Antibodies
[ ]	[x]	Eukaryotic cell lines
[x]	[ ]	Palaeontology
[ ]	[x]	Animals and other organisms
[x]	[ ]	Human research participants
[x]	[ ]	Clinical data

Methods

n/a	Involved in the study	
[x]	[ ]	ChIP-seq
[ ]	[x]	Flow cytometry
[x]	[ ]	MRI-based neuroimaging

Antibodies

Antibodies used

For immunohistochemistry:
Rabbit anti-Cytokeratin 19 (Epitomics AC-0073) 1:200
Rabbit anti-phospho-Stat3 (Tyr705) (CST 9145) 1:100

For flow cytometry:
Chicken anti-c-myc antibody (A21281, Invitrogen) 1:5000
Rabbit anti-6-His-FITC antibody A190-114F, Bethyl Labs) 1:500
Goat anti-chicken Alexa Fluor 488 (A11039, Fisher Scientific) 1:500
Goat anti-chicken phycoerythrin (sc-3730, Santa Cruz Biotechnology) 1:500

Goat anti-chicken Alexa Fluor 647 (NC0928213, Fisher Scientific [Abcam]) 1:500
Goat anti-rabbit Alexa Fluor 488 (A11034, Fisher Scientific) 1:500
Goat anti-mouse Alexa Fluor 488 (A11029, Fisher Scientific) 1:500
Goat anti-mouse Alexa Fluor 647 (A21463, Fisher Scientific) 1:500
Goat anti-human Alexa Fluor 647 (A21445, Fisher Scientific) 1:500

For western blot:
Stat3 (D3Z2G) Rabbit mAb #12640 (NC0969631, Fisher Scientific) 1:5000
phospho-stat3 (pY705) Rabbit mAb (9145S, Fisher Scientific) 1:4000
B-tubulin (TU27/Tubulin) Mouse antibody (903401, BioLegend) 1:5000
LIFR (sc-659, lot 1714, Santa Cruz) 1:200
Peroxidase-AffiniPure Donkey Anti-Mouse IgG (715-035-150, Jackson ImmunoResearch) 1:5000
Peroxidase-AffiniPure Donkey Anti-Rabbit IgG (711-035-152, Jackson ImmunoResearch) 1:5000

For LIF neutralization:
D25 anti-LIF monoclonal antibody (Received from Hunter lab)

Validation

The D25 LIF-neutralizing antibody was previously validated by the Hunter lab (Shi, et. al. Nature 2019; https://doi.org/10.1038/s41586-019-1130-6). All other antibodies were purchased commercially, and websites with their validations are listed below:

For immunohistochemistry:
Rabbit anti-Cytokeratin 19 (Epitomics AC-0073):
https://www.clinisciences.com/en/anti-cytokeratin-7-ce-ivd-for-ihc-4336/rabbit-monoclonal-anti-human-cytokeratin-63006198.html
Rabbit anti-phospho-Stat3 (Tyr705) (CST 9145):
https://www.cellsignal.com/products/primary-antibodies/phospho-stat3-tyr705-d3a7-xp-rabbit-mab/9145

For flow cytometry:
Chicken anti-c-myc antibody (A21281, Invitrogen):
https://www.thermofisher.com/antibody/product/Myc-Tag-Antibody-Polyclonal/A-21281
Rabbit anti-6-His-FITC antibody A190-114F, Bethyl Labs):
https://www.bethyl.com/product/A190-114F
Goat anti-chicken Alexa Fluor 488 (A11039, Fisher Scientific):
https://www.thermofisher.com/antibody/product/Goat-anti-Chicken-IgY-H-L-Secondary-Antibody-Polyclonal/A-11039
Goat anti-chicken phycoerythrin (sc-3730, Santa Cruz Biotechnology):
https://datasheets.scbt.com/sc-3730.pdf
Goat anti-chicken Alexa Fluor 647 (NC0928213, Fisher Scientific [Abcam]):
https://www.abcam.com/goat-chicken-igy-hl-alexa-fluor-647-ab150171.html
Goat anti-rabbit Alexa Fluor 488 (A11034, Fisher Scientific)
https://www.thermofisher.com/antibody/product/Goat-anti-Rabbit-IgG-H-L-Highly-Cross-Adsorbed-Secondary-Antibody-Polyclonal/A-11034
Goat anti-mouse Alexa Fluor 488 (A11029, Fisher Scientific)
https://www.thermofisher.com/antibody/product/Goat-anti-Mouse-IgG-H-L-Highly-Cross-Adsorbed-Secondary-Antibody-Polyclonal/A-11029
Goat anti-mouse Alexa Fluor 647 (A21463, Fisher Scientific)
https://www.thermofisher.com/antibody/product/Chicken-anti-Mouse-IgG-H-L-Cross-Adsorbed-Secondary-Antibody-Polyclonal/A-21463
Goat anti-human Alexa Fluor 647 (A21445, Fisher Scientific)
https://www.thermofisher.com/antibody/product/Goat-anti-Human-IgG-H-L-Cross-Adsorbed-Secondary-Antibody-Polyclonal/A-21445

For western blot:
Stat3 (D3Z2G) Rabbit mAb #12640 (NC0969631, Fisher Scientific)
https://www.cellsignal.com/products/primary-antibodies/stat3-d3z2g-rabbit-mab/12640
phospho-stat3 (pY705) Rabbit mAb (9145S, Fisher Scientific)
https://www.cellsignal.com/products/primary-antibodies/phospho-stat3-tyr705-d3a7-xp-rabbit-mab/9145
B-tubulin (TU27/Tubulin) Mouse antibody (903401, BioLegend)
https://www.biolegend.com/en-us/products/purified-anti-beta-tubulin-antibody-11242
LIFR (sc-659, lot 1714, Santa Cruz)
https://www.scbt.com/p/lifr-antibody-c-19
Peroxidase-AffiniPure Donkey Anti-Mouse IgG (715-035-150, Jackson ImmunoResearch)
https://www.jacksonimmuno.com/catalog/products/715-035-150
Peroxidase-AffiniPure Donkey Anti-Rabbit IgG (711-035-152, Jackson ImmunoResearch)
https://www.jacksonimmuno.com/catalog/products/711-035-152

Eukaryotic cell lines

Policy information about cell lines

Cell line source(s)	The human PDAC cancer cell line PANC1 (CRL-1469) was acquired from ATCC; KP4 (JCRB0182) from JCRB; HeLa STAT3 luciferase reporter cells from Signosis (SI-0003-NP). Both PDAC lines were provided by the Hunter group (Salk), along with the corresponding LIFR knock-down lines. The mouse PDAC line was derived and provided by the Hunter group (Salk) from the KPCL genetic mouse pancreatic cancer model
Authentication	All cell lines were originally authenticated by STR testing by their suppliers. We did not re-authenticate the lines after receiving them.
Mycoplasma contamination	All cell lines were tested regularly for mycoplasma and were negative at the time of use for each experiment.
Commonly misidentified lines (See ICLAC register)	No commonly misidentified cell lines were used.

Animals and other organisms

Policy information about studies involving animals; ARRIVE guidelines recommended for reporting animal research

Laboratory animals	We used immunocompromised female, 6 - 7 week old nude mice (NU/J; 002019; Jackson Laboratory) for the tumor xenograft study. We used female, 5 - 6 week old black/6 (C57BL/6J; 000664; Jackson Laboratory) and friend virus B NIH Jackson (FVB; 001800; Jackson Laboratory) for the toxicity study. KrasLSL-G12D/+;Trp53flox; Rosa26LSL-Luc compound mutant mice (designated as KPf/fL mice) on FVB background were kindly provided by R. Shaw (Salk). Pdx1-Cre mice (stock # 014647) were purchased from The Jackson Laboratory, and backcrossed with KPf/fL mice for at least 6 generations before phenotypic analysis to generate KPf/fCL mice. KPf/fCL mice were 5 - 6 weeks old and a mixture of male and female mice were used.
Wild animals	None.
Field-collected samples	None.
Ethics oversight	Mice were maintained and animal experiments performed in accordance with policies approved by the Stanford University Administrative Panel on Laboratory Animal Care (Protocol no. 33187) and the Salk Institute Animal Care and Use Committee.

Note that full information on the approval of the study protocol must also be provided in the manuscript.

Flow Cytometry

Plots

Confirm that:

- [x] The axis labels state the marker and fluorochrome used (e.g. CD4-FITC).
- [x] The axis scales are clearly visible. Include numbers along axes only for bottom left plot of group (a 'group' is an analysis of identical markers).
- [x] All plots are contour plots with outliers or pseudocolor plots.
- [x] A numerical value for number of cells or percentage (with statistics) is provided.

Methodology

Sample preparation	Yeast were incubated with protein, washed, and stained in 1 g/L bovine serum albumin in phosphate buffered saline (BPBS). Antiobodies were added to detect expression and binding after incubation with target protein. After washing, yeast were spun down and kept on ice until resuspending in BPBS and loading onto the flow cytometer or sorter.
Instrument	A FACSAria IIu (Beckton Dickinson) was used for sorting yeast libraries. A BD Accuri C6 (Beckton Dickinson) was used for standard flow cytometry analysis.
Software	For sorting, data were collected using the FACSDiva (Becton Dickinson) version 8.0.1 software. Other flow cytometry data was collected using the Accuri C6 software (Beckton Dickinson). Data were analyzed in FlowJo version 10.6.1 (Treestar)
Cell population abundance	Purity of sorted cells was determined by flow cytometry. Sorted cells were grown up, and run via flow cytometry for binding. Purity of sorted populations was estimated at >95% in all samples.
Gating strategy	Yeast were first gated for forward scatter vs side scatter (FSC-A vs SSC-A). Next, cells were gated for singlets using forward scatter (FSC-H vs FSC-A). Finally, expressing yeast were identified via c-myc-tag staining, with the c-myc positive population defined by running uninduced and no-secondary controls. In all cases a clear negative and positive population were observed in induced yeast.

- [x] Tick this box to confirm that a figure exemplifying the gating strategy is provided in the Supplementary Information.
